# Development of a High‐Resolution MNP Marker System for Aquatic Biodiversity Monitoring: A Case Study With *Schizothorax prenanti* in the Yangtze River

**DOI:** 10.1111/1755-0998.70063

**Published:** 2025-10-22

**Authors:** Baolong Zhang, Wei Jiang, Zhiwei Fang, Hao Chen, Nan Jiang, Junfei Zhou, Renjing Wan, Sha Li, Tiantian Li, Lu Cai, Huiyin Song, Lun Li, Lifen Gao, Lihong Chen, Hai Peng

**Affiliations:** ^1^ Institute for Systems Biology, School of Life Sciences, Jianghan University Wuhan Hubei China; ^2^ Hubei Key Laboratory of Three Gorges Project for Fish Resource Conservation, Chinese Sturgeon Research Institute of China Three Gorges Corporation Yichang Hubei China; ^3^ National Engineering Research Center of Eco‐Environment in the Yangtze River Economic Belt, China Three Gorges Corporation Wuhan Hubei China; ^4^ State Key Laboratory of Precision Blasting, Jianghan University Wuhan Hubei China

**Keywords:** biodiversity monitoring, conservation genetics, environmental DNA, multiple nucleotide polymorphism, Yangtze River

## Abstract

Effective monitoring of aquatic biodiversity is critical for conservation, yet current approaches such as mitochondrial COI barcoding and microsatellite markers exhibit limitations in resolution, sensitivity, and scalability, particularly for detecting low‐abundance or degraded DNA in mixed aquatic samples. To address these challenges, we developed a novel Multiple Nucleotide Polymorphism (MNP) marker system tailored to *S. prenanti* , an endangered endemic fish species emblematic of biodiversity crises in the Yangtze River. Through restriction‐site associated DNA sequencing, we identified 115 genome‐wide MNP markers. These markers demonstrated ultrasensitive detection (~1 DNA copy/reaction) and high specificity (mean discriminative power = 0.77, calculated as the probability that two random samples differ at a locus). When applied to environmental DNA from the Yangtze River, the MNP system revealed substantial genetic diversity among 86 samples (84% average differentiation rate) and quantified the contribution of artificially stocked fish to natural populations, identified 567 shared alleles between stocked and wild populations. By outperforming traditional methods in analysing fragmented DNA and enabling high‐throughput applications, this MNP framework provides a transformative approach for conservation genetics. Our scalable solution bridges the gap between genetic research and conservation action, offering global applicability for aquatic biodiversity monitoring.

## 1 Introduction

The Yangtze River, Asia's longest and the third‐longest river globally, spans over 6300 km and traverses diverse ecosystems ranging from high‐altitude streams in the Tibetan Plateau to expansive lowland floodplains and estuaries (Chen et al. 2001). This vast hydrological network supports a rich biodiversity, including over 405 species of fish, with nearly half being endemic (He et al. 2020). Historically, the Yangtze River has been a vital cradle for freshwater fisheries, contributing over 70% of China's freshwater fishery production (Liu 1992). However, decades of intense anthropogenic pressures, including dam construction, habitat fragmentation, pollution, and overfishing, have led to drastic declines in fish populations and biodiversity (Li et al. 2020; Liu et al. 2019; Reid et al. 2019). Recognising this alarming trend, the Chinese government implemented the landmark 10‐year fishing ban policy to restore fish stocks and conserve aquatic biodiversity (Wu et al. 2023). Despite this significant measure, monitoring the recovery of fish populations and ensuring effective conservation management remain critical challenges, particularly given the complexities of the Yangtze river's ecosystem and the limitations of traditional ecological survey methods.

Traditional methods, such as morphological identification, are time‐consuming, require expert knowledge, and often fail to differentiate closely related or cryptic species (Kubečka et al. 2009). DNA‐based methods have revolutionised the field of fish species identification, offering more reliable, efficient, and scalable alternatives to traditional morphological approaches (Franco et al. 2021). Among the DNA‐based approaches, mitochondrial COI (cytochrome c oxidase subunit I) barcoding has been the most widely adopted. By analysing a standardised region of the mitochondrial genome, COI barcoding facilitates the rapid identification of a wide range of taxa and has been instrumental in global biodiversity initiatives (April et al. 2011; Durand et al. 2017; Ward et al. 2005). However, this approach faces critical limitations, including its reliance on a single mitochondrial gene, which is maternally inherited and prone to sharing haplotypes among closely related species, thereby reducing taxonomic resolution at finer levels (Shen et al. 2019; Ward et al. 2009). Furthermore, COI barcoding's requirement for > 600 bp amplicons is ill‐suited for degraded environmental DNA (eDNA, fragments typically 150–250 bp) and mainstream NGS platforms (read lengths 150–300 bp), limited its applicability in high‐throughput sequencing‐based biodiversity monitoring (Armani et al. 2015; Chin et al. 2016). Recent adaptations such as mini‐barcoding and metabarcoding have sought to address some of these limitations by targeting shorter DNA fragments (~100–300 bp) or enabling high‐throughput identification of multiple species from mixed eDNA samples (Sultana et al. 2018). However, these methods sacrifice resolution and accuracy when distinguishing between closely related taxa or detecting rare species in low‐concentration samples. Additionally, DNA metabarcoding relies on extensive bioinformatic workflows and dependence on comprehensive reference databases often leads to misidentifications or inconclusive results due to incomplete sequence libraries (Blackman et al. 2024; Naz et al. 2023).

Beyond COI barcoding, nuclear DNA‐based approaches, such as microsatellites (SSR, Simple Sequence Repeats) and single nucleotide polymorphisms (SNPs), offer alternatives for fish identification (Mastrochirico‐Filho et al. 2019). SSR markers, characterised by their high mutation rates and multiallelic nature, have been used extensively in population genetics studies. However, their high susceptibility to amplification slippage and the frequent co‐occurrence of non‐specific bands in mixed DNA samples compromise their reliability in complex eDNA settings (Baptiste and Eckert 2012; Tóth et al. 2000). SNP markers, which target single base‐pair variations in the genome, provide higher specificity and are less prone to amplification errors. Yet, their limited ability to resolve closely related species, coupled with the need for comprehensive reference databases, restricts their applicability for species‐level identification in diverse and understudied ecosystems (Morin et al. 2004). Emerging methodologies such as high‐resolution melting (HRM) analysis and loop‐mediated isothermal amplification (LAMP) have provided new avenues for high‐throughput species identification, yet they still grapple with the challenges of cost, scalability, and handling the complexity of aquatic eDNA samples (Cermakova et al. 2023). These shortcomings underscore the urgent need for a more robust, sensitive, and scalable DNA identification method tailored to the unique challenges of aquatic biodiversity monitoring.

Unlike traditional markers such as SSRs or SNPs, multiple nucleotide polymorphism (MNP) markers, which are similar to microhaplotypes (Baetscher et al. 2018; Kidd and Podini 2024; Osborne et al. 2023), leverage variations at multiple nucleotide positions within short genomic regions (< 300 bp). This multi‐site variation increases allelic combinations, enabling higher discriminative power for distinguishing closely related species, subspecies, and individuals (Fang et al. 2021). Moreover, MNP markers integrate seamlessly with high‐throughput sequencing platforms, enabling the detection of rare or low‐abundance DNA with exceptional sensitivity and accuracy. Like other PCR‐based methods, MNP markers are susceptible to PCR biases and allelic dropout, which can influence the accuracy of genetic analyses (Pompanon et al. 2005). Recent developments in MNP genotyping methods, such as MNP‐Seq, have demonstrated remarkable efficiency and precision, achieving near‐perfect accuracies in both diploid and polyploid systems (Ling et al. 2022; Liu et al. 2023; Liu, Wang, et al. 2024; Liu, Zhao, et al. 2024; Yi et al. 2024). These attributes suggested that MNP technology could offer a robust and scalable approach to address the unique challenges posed by aquatic eDNA samples. By potentially incorporating ultra‐multiplex amplification and advanced bioinformatics pipelines, MNP‐based systems might provide a comprehensive and reliable framework for aquatic biodiversity monitoring, thereby advancing our ability to assess ecosystem health and inform conservation efforts.

In this study, we developed a novel MNP marker system tailored for *Schizothorax prenanti* , an endemic Yangtze River fish of significant ecological and economic importance (Qin et al. 2016). As a key species in high‐altitude stream ecosystems, *S. prenanti* supports food webs and local fisheries but has faced severe population declines due to habitat fragmentation and overfishing (Dudgeon 2010). Prior genetic studies using mitochondrial markers revealed limited population structure but highlighted the need for higher‐resolution tools to monitor its conservation status (Ma et al. 2020; Song et al. 2008). By surpassing the limitations of traditional DNA‐based methods, this work establishes a scalable framework for aquatic biodiversity research, aligning with conservation initiatives such as the Yangtze River fishing ban and offering potential applications across diverse ecosystems worldwide.

## 2 Materials and Methods

### 2.1 *S. prenanti* Samples and DNA Extraction

Genomic DNA samples were obtained from 36 *S. prenanti* individuals sourced from the Chinese Sturgeon Research Institute of China Three Gorges Corporation: 30 from wild populations in the Yangtze River and 6 from hatchery‐reared fish (stocked populations). The identification of *S. prenanti* was confirmed using molecular methods targeting the mitochondrial cytochrome c oxidase subunit I (*COI*) and cytochrome b (*CYTB*) genes (Hebert et al. 2003; Ma et al. 2020). Individuals were classified as *S. prenanti* only if both *COI* and *CYTB* sequences matched the reference sequences for this species. Water samples were collected from six locations along the Yangtze River: Chishui River Port in Hejiang (CSHK‐HJ), downstream of Chishui River Port in Hejiang (CSHK‐XY‐HJ), upstream of Luzhou (LZ‐SY), downstream of Luzhou (LZ‐XY), Jinsha River Public Security Area in Yibin (JSJ‐SSGA‐YB), and downstream of Xiangjiaba Dam in the Jinsha River, Yibin (JSJ‐XJB‐YB). At each site, 8–10 water samples (1 L each) were collected, yielding a total of 56 eDNA samples (Table 1).

**TABLE 1:** Environmental DNA (eDNA) water sampling site information.

Site	Number of samples	Latitude (°N)	Longitude (°E)	Location description
CSHK‐HJ	8	28.803	105.845	Chishui River Port, Hejiang
CSHK‐XY‐HJ	9	28.824	105.872	Downstream of Chishui River Port
LZ‐SY	10	28.862	105.413	Upstream of Luzhou
LZ‐XY	10	28.875	105.512	Downstream of Luzhou
JSJ‐SSGA‐YB	9	28.785	104.753	Jinsha River, Public Security Area
JSJ‐XJB‐YB	10	28.641	104.408	Downstream of Xiangjiaba Dam

Total genomic DNA was extracted from fins tissues of the collected *S. prenanti* using the TIANamp Genomic DNA Kit (Tiangen Biotech, Beijing, China) following manufacturer guidelines. Briefly, approximately 20 mg of fins tissue was homogenised, lysed using proteinase K, and subjected to spin‐column‐based DNA purification. At each of the six sampling sites, 8–10 replicate 1 L water samples were filtered on‐site within 2 h of collection using a 0.22 μm nitrocellulose filter membrane to concentrate DNA. Filters were immediately stored at −20°C in sterile tubes containing 95% ethanol to minimise DNA degradation (Turner et al. 2015). Filters were transported to the laboratory within 24 h and stored at −80°C until DNA extraction. eDNA was extracted using the Jianshi DNA Extraction Kit for Water Samples (Jianshi Biotech, Wuhan, China).

The DNA copy number of *S. prenanti* was calculated based on the DNA concentration and the genome size of *S. prenanti* (~1Gb) based the following formula:

$$\begin{array}{c} \text{Copy number}\;\left(\text{copies}/\mathrm{\mu L}\right)=\mathrm{DNA}\;\text{concentration}\;\left(\mathrm{ng}/\mathrm{\mu L}\right)\\ \times 9.12\times 10^{11}/\text{genome size}\;\left(\mathrm{bp}\right). \end{array}$$

where 9.12 × 10^11^ was a constant derived from Avogadro's number (6.022 × 10^23^) and the average molecular weight of double‐stranded DNA (660 g/mol/bp).

### 2.2 Restriction‐Site Associated DNA Sequencing

Restriction‐site associated DNA sequencing (RAD‐seq) libraries were prepared followed the standardised protocol, with minor modifications (Liu, Zhao, et al. 2024). In brief, Genomic DNA from *S. prenanti* was enzymatically digested using MseI and EcoRI‐HF, followed by adapter ligation with P5 and P7 Illumina‐compatible barcodes. Ligation products were purified using magnetic beads and size‐selected to enrich for fragments in the 100–300 bp range. The libraries were PCR‐amplified using 12 cycles, quantified using a Qubit Fluorometer (Thermofisher, USA), and subjected to quality control using 1% agarose gel electrophoresis.

### 2.3 Screening and Primer Design for MNP Markers in *S. prenanti*

MNP marker development was based on the RAD‐seq data from 30 representative *S. prenanti* . High‐quality sequencing reads were generated after removing low‐quality bases (quality score < 20) and adapter sequences, followed by de novo assembly using Stacks software (Rivera‐Colón and Catchen 2022). The resulting draft assembly provided a foundational reference for the identification of candidate genomic regions harbouring MNPs.

**FIGURE 1:**
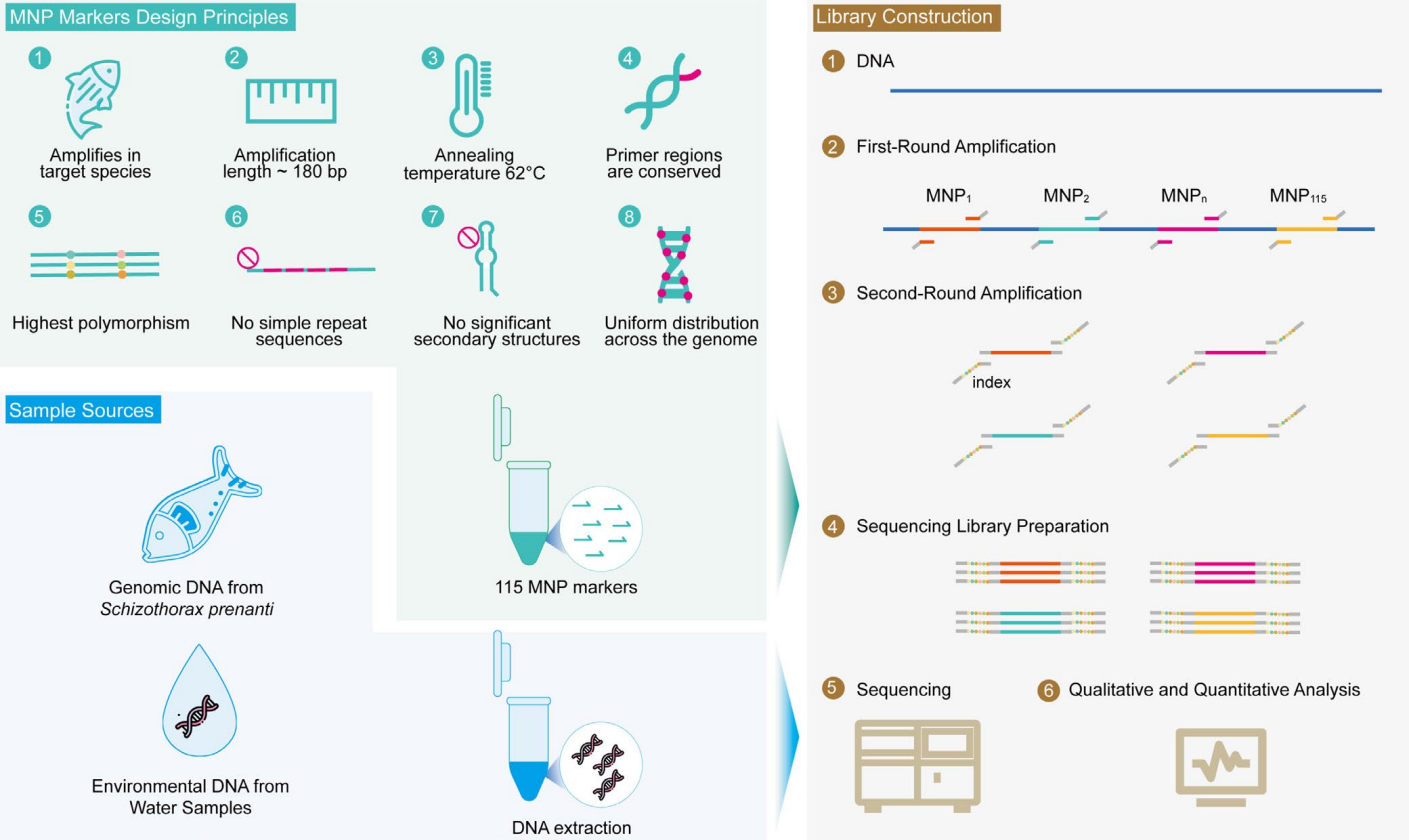
Workflow of MNP Marker Development for *S. prenanti* . Schematic representation of the overall workflow for developing MNP markers for *S. prenanti* . The process involves marker design based on specific criteria (e.g., species specificity, polymorphism, length, and distribution), and MNP library preparation. The MNP library construction includes two rounds of PCR amplification: (1) multiplex PCR to amplify 115 target markers and (2) barcode and adapter addition for high‐throughput sequencing.

To design MNP markers, a sliding window approach was applied, using overlapping genomic windows of 120 base pairs (bp) with a 5 bp increment to screen for multiple dispersed SNPs. Overlapping windows ensured comprehensive coverage of polymorphic regions. Each window's discriminative power (DP) was calculated as DP = *n*/*N*, where ‘*n*’ is the number of sample pairs with at least three SNPs in a given window, and ‘*N*’ is the total number of sample pairs compared. Windows with DP > 0.2 were prioritised to optimise resolution, based on pilot analyses showing robust differentiation at this threshold (Fang et al. 2021). The stringent selection criteria for MNP markers were as follows: each marker was polymorphic, and approximately 180 bp in length, ensuring high resolution and reliability. The MNP markers were distributed evenly across the genome to provide comprehensive genetic coverage (Figure 1). Primers flanking the selected MNP loci were designed using Primer3 software. Specifically, Primers were standardised to a uniform annealing temperature of 62°C, avoided simple repeat sequences, and exhibited no secondary structure.

### 2.4 Cross‐Species Specificity Testing of MNP Markers

Three wild *S. davidi* individuals were collected, and genomic DNA was extracted using the TIANamp Genomic DNA Kit (Tiangen Biotech, Beijing, China). Species identity was confirmed via *CYTB* and *COI* sequencing (Ma et al. 2020). Cross‐amplification was assessed by detection rates of MNP loci, and genetic differentiation was calculated as the proportion of differing MNP loci between and within species. For eDNA applications, species‐specific ‘Core Alleles’ were identified by comparing allele frequencies across all 115 loci in *S. prenanti* (*n* = 30) and *S. davidi* (*n* = 3). Core Alleles were defined as those with ≥ 90% frequency in one species and < 10% in the other.

### 2.5 Synthetic DNA Spike‐Ins

Synthetic DNA spike‐ins were developed as external standards to facilitate quantification of target DNA during MNP‐seq (Gao et al. 2023). Each DNA spike‐in was synthetically designed to meet stringent criteria, including balanced GC content and no homology with known sequences in public databases. These synthetic sequences ensured no cross‐reactivity with *S. prenanti* or environmental DNA. Three synthetic spike‐ins were synthesised, containing 6, 7, and 7 MNP loci, respectively, for a total of 20 loci (Table S1). The number of loci per spike‐in was chosen to balance sequencing coverage and cost, with 6–7 loci providing sufficient redundancy for accurate quantification across a range of DNA concentrations. The DNA spike‐ins were added to each sample prior to library preparation, ensuring uniformity across experimental replicates. The DNA spike‐in based copy number of *S. prenanti* DNA in each reaction was calculated using the following formula:

$$\text{Copy number}=C_{s}/C_{d}\times 55500$$

where *Cs* is the average sequencing coverage of 115 *S. prenanti* MNP loci, and *C*_*d*_ is the average coverage of 20 DNA spike‐in MNP loci in each reaction. The constant 55,500 represents the copy number of spike‐in DNA (5.55 × 10^4^ copies per reaction), added as an external standard to calibrate *S. prenanti* DNA quantification. Synthetic DNA spike‐ins were synthesised by Sangon Biotech (Shanghai). The nominal number of synthetic spike‐in copies added to each PCR reaction (5.55 × 10^4^ copies per reaction) was supplied on the vendor certificate of analysis.

### 2.6 Library Construction and MNP Sequencing

The MNP‐Seq libraries construction process included two sequential PCR amplification steps. In the first round, we performed multiplex PCR using 115 primer pairs to target specific MNP loci of *S. prenanti* . The second round of PCR added barcodes for sample differentiation and adapters compatible with high‐throughput sequencing platforms (Figure 1). DNA libraries were constructed using protocols for targeted multiplex PCR‐based protocol with minor adjustments (Gao et al. 2023; Ling et al. 2022). Briefly, DNA (200 ng) was amplified with a custom primer panel targeting 115 loci, followed by size selection of 200–500 bp fragments using magnetic beads. Amplified products were further processed to include sequencing adapters and barcodes. Quality control included agarose gel electrophoresis and fluorometric quantification, ensuring library integrity and concentrations above 10 ng/μL. Equimolar pooling of libraries was performed before sequencing on an Illumina platform with paired‐end 150 bp reads.

### 2.7 The Reproducibility and Accuracy of the MNP Marker System

Ten *S. prenanti* samples were randomly selected and performed two independent DNA extractions per sample. Two sequencing libraries (Library 1 and Library 2) were constructed for each sample using the multiplex PCR protocol. Genotyping results from both libraries were compared, with precision (*r*) calculated as the proportion of loci with identical genotypes (M1) to the total loci detected in both libraries (M2), i.e., r=M1/M2. Accuracy (*a*) was derived as $a=1-\left(1-r\right)/2$.

### 2.8 Data Analysis

MNP call frequencies for each marker were determined by calculating the proportion of samples in which the MNP locus was detected. The proportion of heterozygous individuals for each locus was determined by calculating the proportion of individuals that exhibited a heterozygous genotype at that locus. Phylogenetic tree was constructed using the Neighbour‐Joining (NJ) method, which is commonly employed for inferring evolutionary relationships based on genetic distance (Kamvar et al. 2014).

## 3 Results

### 3.1 Development of MNP Markers for *S. prenanti*

**FIGURE 2:**
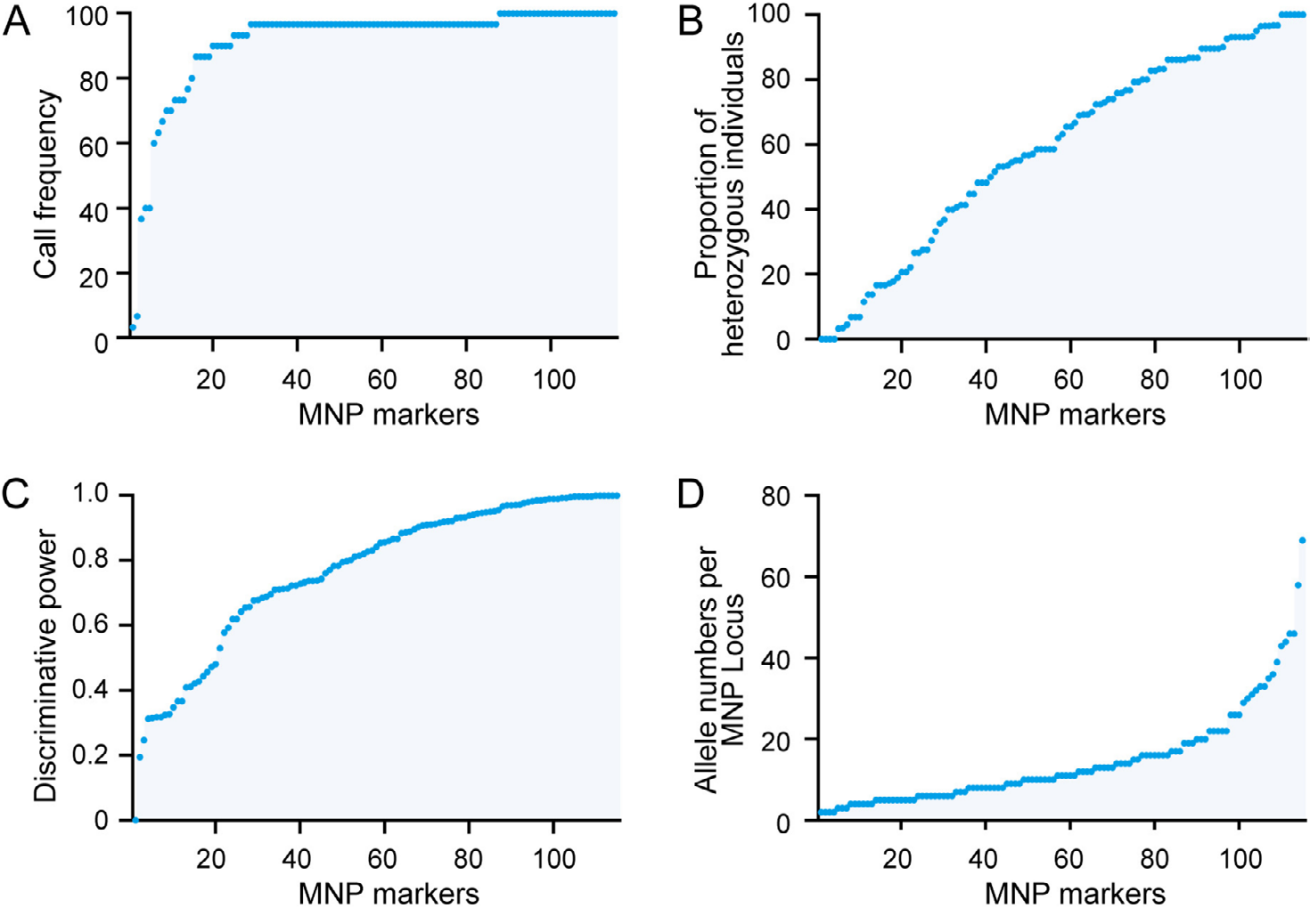
Detection and Evaluation of MNP Markers in *S. prenanti* . (A) Distribution of MNP call frequencies across 30 samples. (B) The proportion of heterozygous individuals for each MNP marker. (C) The discriminative power of 115 MNP loci across 30 samples. (D) Number of allele genotypes per MNP marker.

While RAD‐seq identified over 400 potential MNP loci, we selected 115 based on cost‐effectiveness and experimental feasibility, ensuring sufficient resolution for biodiversity monitoring (Table S2). To evaluate the performance of these MNP markers, we analysed their detection rates, the proportion of heterozygous individuals, discriminative power, and allele diversity across 30 *S. prenanti* (Figure 2). The call frequency exceeded 90% for 96 markers (83.5%) and surpassed 30% for 113 markers (98.3%), demonstrating the high reliability of the markers in detecting target DNA (Figure 2A). The proportion of heterozygous individuals ranged from 0% to 100%, with an average of 59.1%, reflecting substantial genetic diversity captured by the markers (Figure 2B). The mean discriminative power of the markers was 0.77, and 113 markers (98.3%) exhibited discriminative power above 0.2, indicating their effectiveness in distinguishing genetic differences among samples (Figure 2C). Furthermore, a total of 1680 allele genotypes were identified, with an average of 14.6 alleles per marker, underscoring the system's capacity for fine‐scale genetic resolution (Figure 2D). Additionally, results of the reproducibility and accuracy showed that the system achieved 100% reproducibility and accuracy across 10 samples, confirming its reliability for genotype calling (Table S3). These results validate the effectiveness and robustness of these 115 MNP markers, provided a solid foundation for downstream applications in fish DNA identification and biodiversity monitoring.

### 3.2 Validation of MNP Marker Specificity

**FIGURE 3:**
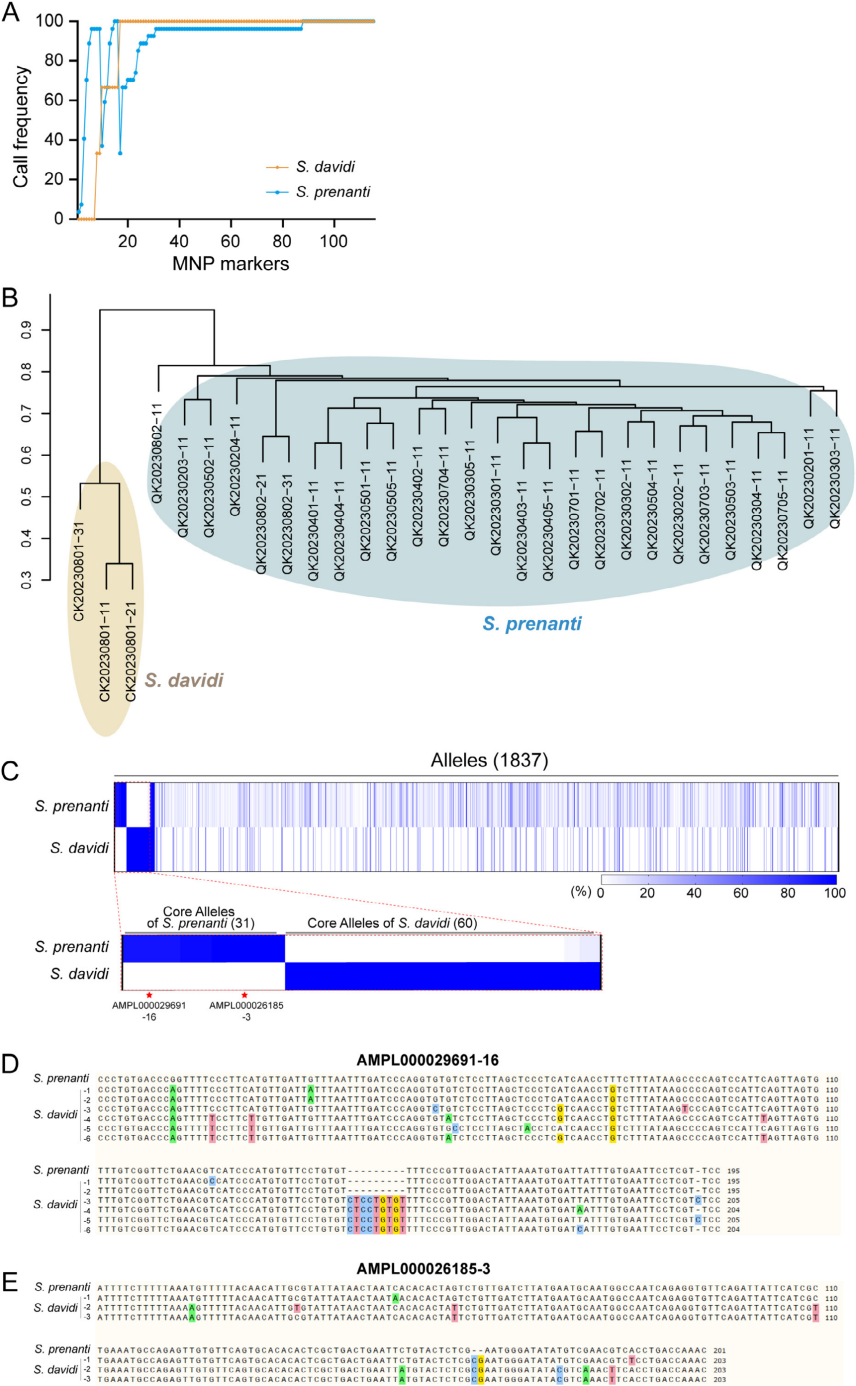
Validation of MNP Marker Specificity in *S. prenanti* and *S. davidi* . (A) MNP call frequencies across *S. prenanti* and *S. davidi* . (B) Neighbour‐joining genetic clustering of *S. prenanti* and *S. davidi* . Phylogenetic tree showing genetic differentiation based on MNP loci. (C) Alleles identified in *S. prenanti* and *S. davidi* . Number of the total alleles detected and species‐specific Core Alleles in our validation dataset for each species was shown. (D, E) SNP differences for AMPL000029691‐16 (D) and AMPL000026185‐3 (E) between Core Alleles of *S. prenanti* and all *S. davidi* alleles. The positions of differentially represented SNP loci are colour‐coded in four distinct colours.

To evaluate the specificity of the MNP marker, we tested its amplification in three *Schizothorax davidi* individuals, a closely related species (Yan et al. 2017). Of the 115 markers, 86% (*n* = 99) amplified consistently across all the three *S. davidi* samples, whereas 6% (*n* = 7) failed to amplify (Figure 3A). Despite this high level of cross‐amplification, genetic differentiation between the two species remained distinct. Neighbour‐joining clustering based on MNP loci effectively separated *S. prenanti* from *S. davidi* in tissue samples, with interspecific differentiation rates exceeding 90%, compared to 60%–80% within *S. prenanti* and 30%–50% within *S. davidi* (Figure 3B).

For reliable detection in eDNA samples, where mixed DNA may obscure species identification, we identified species‐specific Core Alleles in our validation dataset (defined as alleles with a frequency ≥ 90% in one species and < 10% in the other). Analysis of *S. prenanti* and *S. davidi* samples revealed 1837 alleles across 115 loci, from which 31 *S. prenanti* ‐specific and 60 *S. davidi* ‐specific Core Alleles were identified (Figure 3C; Table S3). These Core Alleles displayed 3–8 SNP differences between the species, as demonstrated by AMPL000029691‐16 (Figure 3D) and AMPL000026185‐3 (Figure 3E). This robust genetic distinction enables accurate identification of *S. prenanti* in mixed eDNA samples, minimising the risk of false positives from closely related species.

### 3.3 Sensitivity of MNP Markers for *S. prenanti* DNA Identification

**FIGURE 4:**
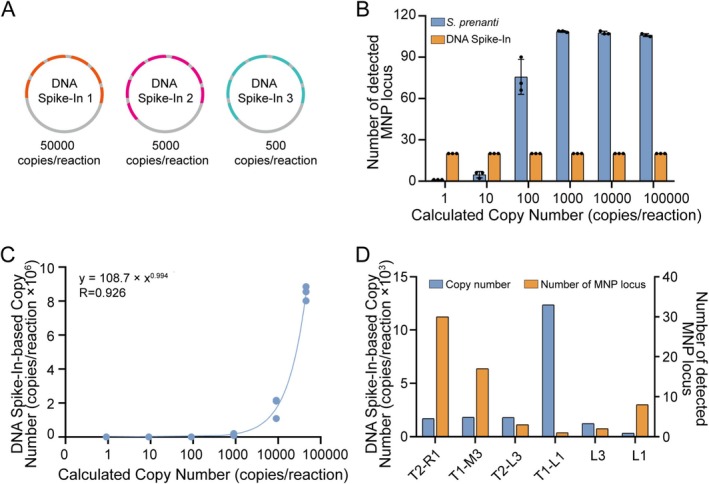
Sensitivity evaluation of the DNA identification system for *S. prenanti* using DNA spike‐ins. (A) Diagram of the three DNA spike‐ins designed for the sensitivity analysis. DNA spike‐in 1, DNA spike‐in 2, and DNA spike‐in 3 contain 6, 7, and 7 MNP loci, respectively, with a total of 20 loci. (B) Quantification of MNP loci detected for DNA spike‐ins and *S. prenanti* in six experimental groups with different DNA concentrations. (C) Calibration curve for the detection of *S. prenanti* DNA, demonstrating the linear relationship between *S. prenanti* DNA concentration (*x*‐axis) and the DNA Spike‐In‐based copy number (*y*‐axis). (D) Detection of *S. prenanti* in six environmental DNA samples, with the number of detected MNP loci and estimated copy numbers presented for each sample.

DNA spike‐ins were employed as external standards to quantification of *S. prenanti* DNA. DNA spike‐ins were synthetic DNA fragments with known copy numbers that enable the calculation of target DNA concentration by comparing their sequencing coverage to that of the target DNA (Blackburn et al. 2019; Gao et al. 2023). In this study, three DNA spike‐ins were designed, each containing 6, 7, and 7 MNP loci, respectively (a total of 20 loci; Figure 4A).

To determine the detection sensitivity of the system, *S. prenanti* DNA was serially diluted to six concentrations: 0.001, 0.01, 0.1, 1, 10, and 50 ng/reaction (0.912, 9.12, 91.2, 912, 9120, and 45,600 copies/reaction). Our results showed that all 20 DNA spike‐in MNP loci were detected in each of the six experimental groups. The number of detected *S. prenanti* MNP loci increased proportionally with DNA concentration, averaging 1, 5, 76, 109, 108, and 106 loci for the respective concentration groups (Figure 4B). Notably, even at the lowest concentration (~1 copy/reaction), at least one *S. prenanti* MNP locus was detected, demonstrated that the system can reliably detect DNA at concentrations above ~1 copy/reaction (Figure 4C). Such sensitivity ensures the system's utility in detecting low‐abundance DNA, a key requirement for eDNA‐based monitoring in aquatic environments.

To test the system's real‐world applicability, we analysed six eDNA samples randomly collected from aquatic habitats. Results showed that a range of *S. prenanti* MNP loci were detected from 1 to 30, corresponding to estimated DNA copy numbers between 317 and 1694 per reaction (Figure 4D). These findings confirm the system's robustness in detecting *S. prenanti* DNA in complex environmental samples, even when DNA is highly fragmented or present at low concentrations.

### 3.4 Genetic Differentiation and Clustering of *S. prenanti* Based on MNP Markers

To evaluate the utility of the MNP marking system in biodiversity studies, we analysed 86 DNA samples, comprising 30 *S. prenanti* genomic DNA samples and 56 eDNA samples collected from the Yangtze River. Using MNP markers, we calculated pairwise genetic differentiation rates and conducted clustering analyses to assess genetic diversity and population structure.

**FIGURE 5:**
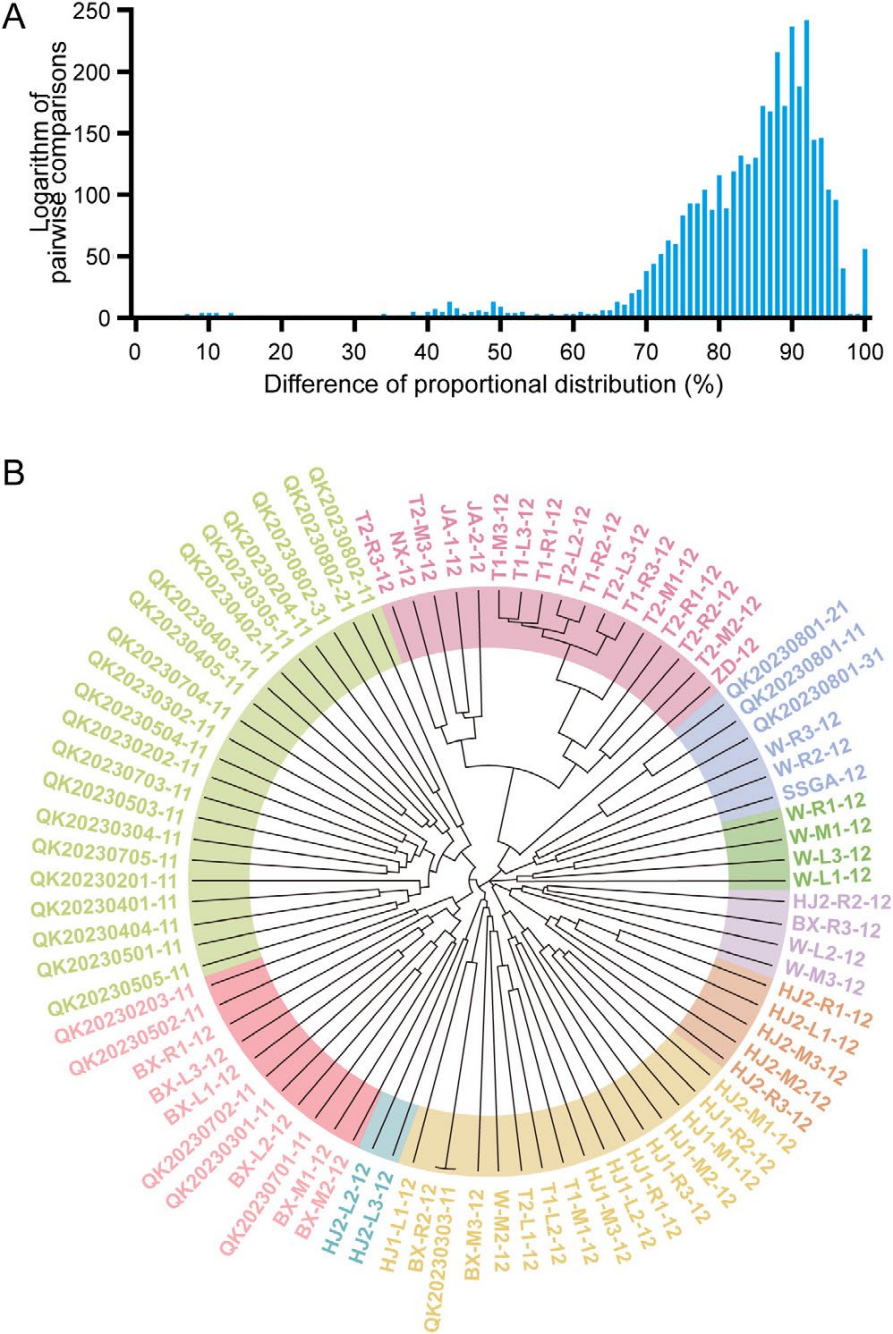
Genetic differentiation and clustering analysis of 86 DNA samples using the MNP marking system. (A) Pairwise genetic differentiation rates calculated from MNP loci across 3655 sample pairs. The genetic differentiation rate is defined as the proportion of differing MNP loci relative to the shared MNP loci between two samples. (B) Neighbour‐joining genetic clustering of the 86 samples based on MNP difference rates. Thirty *S. prenanti* genomic DNA samples are labelled as “QK2023”, while the remaining 56 samples are environmental DNA from the Yangtze River.

A total of 3655 pairwise comparisons were performed across 86 samples (30 genomic DNA from *S. prenanti* and 56 eDNA samples) to quantify genetic differentiation. The genetic differentiation rate, defined as the proportion of differing MNP loci relative to shared loci between two samples, averaged 84% (with stratified values of 79.3% for tissue‐tissue comparisons, 86.6% for tissue‐eDNA, and 78.1% for eDNA‐eDNA pairs) (Figure 5A). Based on the calculated genetic differentiation rates, a neighbour‐joining genetic clustering analysis grouped the 86 samples into nine distinct clusters (Figure 5B). The *S. prenanti* genomic DNA samples, clustered separately from most eDNA samples, reflecting their unique genetic signatures. Notably, eDNA samples from the same site (e.g., LZ‐SY, JSJ‐XJB‐YB) often clustered together, suggesting spatial genetic structure, though some sites (e.g., CSHK‐HJ) showed greater dispersion, possibly due to DNA degradation or lower *S. prenanti* abundance. These results underscore the system's resolution in distinguishing genetic variation across samples, making it well‐suited for analysing biodiversity in complex aquatic environments.

### 3.5 Evaluation of Artificial Stocking Effectiveness Using the *S. prenanti* MNP Marking System

**FIGURE 6:**
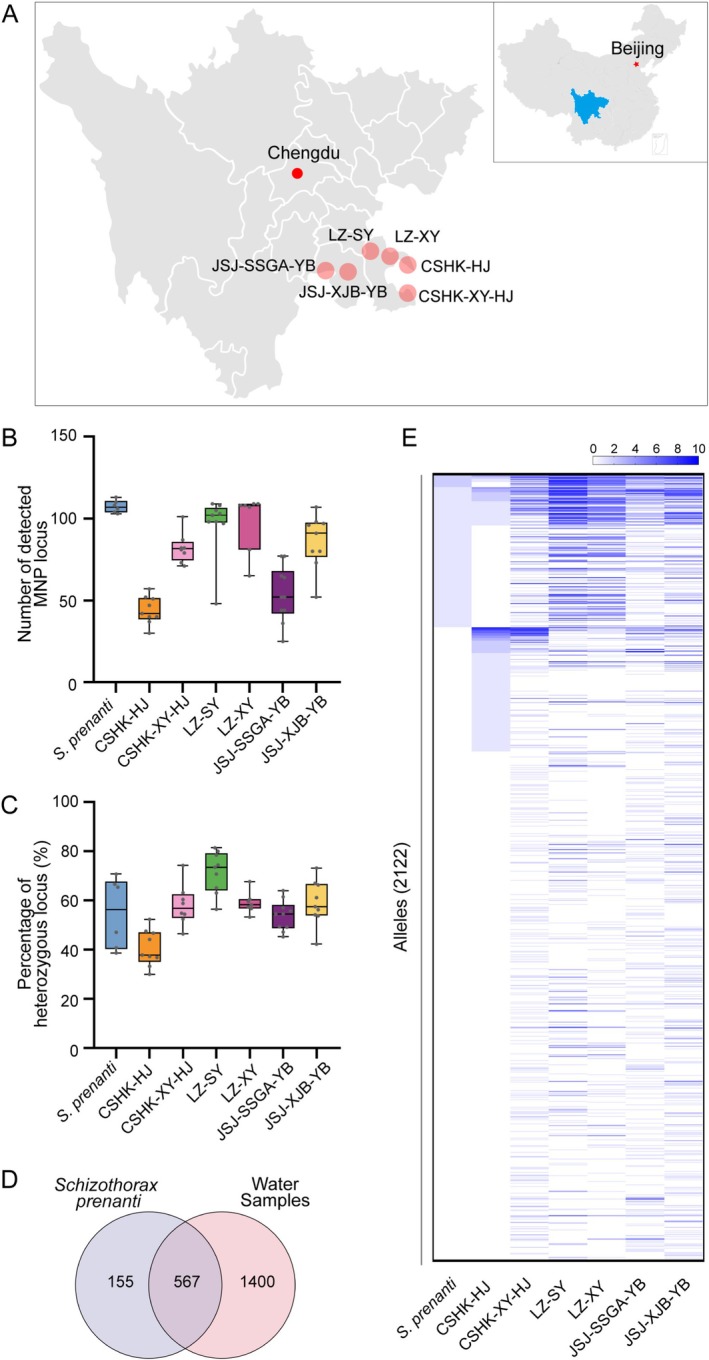
Application of the *S. prenanti* MNP marking system to evaluate artificial stocking outcomes in the Yangtze River. (A) Sampling locations of 56 environmental DNA samples collected from six sites in the Yangtze River: CSHK‐HJ (Chishui River port, Hejiang), CSHK‐XY‐HJ (downstream of Chishui River port, Hejiang), LZ‐SY (upstream of Luzhou), LZ‐XY (downstream of Luzhou), JSJ‐SSGA‐YB (Jinsha River, Public Security Area, Yibin), and JSJ‐XJB‐YB (downstream of Xiangjiaba Dam, Jinsha River, Yibin). (B) Number of detected MNP loci in *S. prenanti* samples prepared for release and environmental samples. (C) Percentage of heterozygous loci in *S. prenanti* samples prepared for release and environmental samples. (D) Comparison of detected alleles. (E) Spatial distribution of shared alleles across sampling locations. The colour intensity gradient in the heatmap corresponds to the spatial distribution frequency (sample count) of specific alleles across distinct geographical regions.

Artificial stocking programs are widely implemented as a conservation strategy to restore fish populations in degraded aquatic ecosystems. These programs involve the release of hatchery‐reared fish into natural habitats to augment wild populations, enhance genetic diversity, and support ecosystem recovery. However, accurately evaluating the effectiveness of these programs remains a persistent challenge (Naish et al. 2007; Trushenski et al. 2010). To address these challenges, we applied this system to evaluate the genetic contribution of released fish and assess biodiversity patterns across six locations in the Yangtze River (Figure 6A). Our results showed that released fish samples exhibited an average detection of 107 MNP loci (93.3%), whereas environmental samples showed a significantly lower detection of 77 loci (66.7%). Notably, MNP detection displayed substantial spatial variation across the six sampling sites, with the CSHK‐HJ site recording the minimum detection of only 44 loci, reflected DNA degradation in eDNA samples (Figure 6B). The percentage of heterozygous loci was 55% in released fish samples and slightly higher, at 57%, in environmental samples (Figure 6C). This consistency suggested that the genetic diversity of the released fish aligns closely with that of the natural population, highlighting the genetic robustness of the artificial stocking process.

We detected 722 alleles in released fish samples and 1967 alleles in environmental samples, with 567 alleles shared between the two groups (Figure 6D). The overlap of alleles indicates that released fish contribute significantly to the natural population. Conversely, the presence of unique alleles in environmental samples suggested additional biodiversity in the river system beyond the released fish. The distribution of shared alleles revealed substantial integration of released fish alleles across five sites: CSHK‐XY‐HJ, LZ‐SY, LZ‐XY, JSJ‐SSGA‐YB, and JSJ‐XJB‐YB. However, CSHK‐HJ exhibited fewer shared alleles, suggested a lower presence of released fish in this region (Figure 6E). Taken together, the *S. prenanti* MNP marker system provided a precise and scalable method to quantify the contribution of released fish to natural populations and evaluate biodiversity patterns in the Yangtze River.

## 4 Discussion

In this study, we developed a set of MNP markers to facilitate precise species identification and genetic analysis of *S. prenanti* . For clarity, while MNPs overlap conceptually with short multi‐SNP “microhaplotypes”, here we use “MNP” to denote loci that were operationally selected by our pipeline (polymorphism density and discriminative‐power thresholds, primer and multiplexability filters, and constrained amplicon lengths) specifically to maximise robustness in high‐multiplex PCR and degraded‐DNA applications. The MNP markers exhibit high call frequencies, averaging over 90% (Figure 2A), and offer substantial allele diversity with an average proportion of heterozygous individuals of 59.1% (Figure 2B), suggested that the MNP system is robust and reliable for distinguishing closely related taxa. Additionally, our sensitivity tests revealed that these markers could detect low concentrations of DNA as minimal as ~1 copy/reaction (Figure 4C). The genetic analyses conducted using these MNP markers provided significant insights into the population dynamics of *S. prenanti* , revealing high genetic differentiation among samples, with a mean value of 84% (Figure 5A). These results not only address the methodological limitations encountered in previous studies but also highlight the potential of MNP markers to unravel the genetic intricacies of aquatic ecosystems.

### 4.1 Enhanced Sensitivity Capabilities of MNP Markers in eDNA Analysis

In natural environments, teleost fishes continuously shed DNA through epithelial cells, mucus, and metabolic waste, with studies estimating that a single fish may release up to 10^4^–10^5^ copies of mitochondrial DNA per day into the water (Itakura et al. 2019; Takahara et al. 2012). However, environmental stressors—including enzymatic degradation, ultraviolet radiation, and microbial activity—rapidly fragment eDNA into short segments (< 200 bp) and reduce its persistence to mere hours in flowing ecosystems (Harrison et al. 2019; Jo et al. 2017). In large river basins like the Yangtze, hydrodynamic dispersion further dilutes eDNA to trace concentrations, rendering conventional methods reliant on long amplicons or high DNA integrity ineffective for species detection (Beng and Corlett 2020).

Our MNP system overcomes these limitations through synergistic design principles. Firstly, short amplicons (180 bp) align with the dominant size range of degraded eDNA fragments, enabling the detection efficiency of the MNP markers underscores their reliability in identifying *S. prenanti* . Specifically, 30 *S. prenanti* genomic DNA samples exhibited an average detection of 107.33 MNP loci, with an exceptionally high mean detection ratio of 93.33%. In contrast, 56 eDNA samples from the Yangtze River demonstrated an average detection of 77.02 loci with a mean detection ratio of 66.97%, reflected DNA degradation in eDNA samples (Figure 5). Despite this reduction, the probability of detecting at least one marker in eDNA samples is mathematically guaranteed at 100.00%. The probability of detecting more than *n*− markers from 115 markers is *P*(*n*), ($P\left(n\right)=\sum_{k=n}^{115}\left(\begin{array}{c} 115\\ k \end{array}\right)66.97{\%}^{k}{\left(1-66.97\%\right)}^{115-k}$, and $P\left(n\geq 1\right)=1-{\left(1-66.97\%\right)}^{115}\approx 100\%$), highlighting the unique capacity of the MNP system to address challenges associated with degraded DNA in aquatic samples. In addition, we observed that the standard deviation of detection ratios in eDNA samples (21.96%) is 7.32 times higher than that of genomic DNA samples (3.00%), suggested significant variability in DNA abundance and degradation levels among different eDNA samples. Secondly, the MNP marker system demonstrates exceptional sensitivity in the quantification of *S. prenanti* DNA in eDNA samples. By incorporating external DNA spike‐ins as quantitative references, DNA copy numbers were estimated with high accuracy and high sensitivity (~1 copy/reaction) (Figure 4A–C). Across six tested eDNA samples, the average *S. prenanti* DNA copy number was 3204, with a maximum of 12,372 copies and a minimum of 317 copies per reaction (Figure 4D), these results underscored its capability to detect and quantify low‐abundance eDNA. However, developing hundreds of MNP markers via RAD‐seq entails significant upfront costs, including sequencing and bioinformatic expertise, which may be prohibitive for some species or labs. To adapt the method for resource‐limited settings, fewer markers (e.g., 20–30 loci) could be prioritised based on high discriminative power (DP > 0.5). For species lacking genomic resources, alternative approaches like amplicon sequencing of conserved regions or de novo SNP discovery via low‐coverage whole‐genome sequencing could generate candidate MNP loci at lower cost.

### 4.2 Assessing Genetic Contributions From Artificial Stocking Using MNP Markers

Accurate differentiation between closely related species and degraded eDNA samples is a persistent challenge in aquatic biodiversity research (Cilleros et al. 2019; Joseph et al. 2022). Our MNP marker system demonstrates superior discriminative power, with a mean discriminative value of 0.77 across all markers (Figure 2C). Additionally, the genetic differentiation rate between 30 *S. prenanti* genomic DNA samples and 56 eDNA samples reached an average of 84% (Figure 5A). This high rate reflected the composite nature of eDNA samples, which capture DNA from multiple individuals or subpopulations across diverse habitats, as well as the high resolution of MNP markers.

The potential for false positives in eDNA due to cross‐amplification in related species like *S. davidi* was a key concern. Our validation shows that, while 86% of markers amplify across species, sequence variation ensures differentiation in tissue samples. For eDNA, Core Alleles enhance specificity, though validation with only three *S. davidi* samples limits generalizability. Other *Schizothorax* species in the Yangtze River may share alleles, requiring broader testing. These limitations highlight the need for future studies with increased sampling to refine the panel's eDNA applicability.

Notably, our method shares conceptual parallels with recent advances in eDNA‐based population quantification, such as Ai et al. (2024) use of segregating mitochondrial sites to estimate species abundance. Both methodologies address the limitations of conventional eDNA concentration metrics by leveraging genetic heterogeneity within evolving populations. However, the systems differ fundamentally in operational scale and resolution. While Ai et al. (2024) utilised mitochondrial sequence polymorphisms from 11 pre‐selected loci to infer fish numbers in controlled mesocosms, our MNP markers target nuclear genomic variations (115 loci) specifically optimised for tracking *S. prenanti* in complex riverine environments.

This nuclear focus provides critical advantages when assessing artificial stocking outcomes in large, biodiverse ecosystems such as the Yangtze River. Artificial stocking programs are central to conservation efforts in the Yangtze River, yet their effectiveness has historically been difficult to evaluate due to the inability to distinguish between stocked and natural populations (Molony et al. 2003). Existing methods for evaluating stocking success, such as mark‐recapture techniques, otolith microchemistry, and traditional genetic analysis, have notable limitations (Cadrin et al. 2013). Mark‐recapture studies are labor‐intensive, logistically challenging in large river systems, and limited to short‐term evaluations (Zhu et al. 2017). Otolith microchemistry requires invasive sampling and provides limited information on genetic integration (Xuan et al. 2022). Traditional genetic approaches using SSRs or mitochondrial markers often lack the resolution and scalability needed for large‐scale biodiversity assessments (Mastrochirico‐Filho et al. 2019; Shen et al. 2019). Our study provides a solution to this challenge, and we identified 1400 unique alleles specific to natural *S. prenanti* populations (eDNA) and 155 unique alleles specific to stocked populations (Figure 6D). This genetic distinction offers unprecedented precision in evaluating key aspects of stocking programs, including the survival and integration of stocked individuals, their reproductive success, and potential hybridization with natural populations. The capacity to simultaneously address species differentiation, population dynamics, and stocking effectiveness establishes the MNP marker system as a transformative tool for aquatic conservation.

### 4.3 Addressing Technical Challenges and Scaling MNP Applications in Aquatic Ecosystems

Although many loci showed exclusive occurrence of particular core alleles in *S. prenanti* within our samples, cross‐species testing was limited (notably *S. davidi* , *n* = 3). Consequently, we restrict claims of “species‐specificity” to our validation dataset and recommend expanding empirical testing to include additional congeners sampled across the Yangtze drainage. We provide an explicit, conservative decision rule here as an operational recommendation for practical monitoring: a water sample is scored positive for *S. prenanti* when (1) at least three core putative *S. prenanti* ‐specific MNP alleles are detected, and (2) each detected allele is supported by ≥ 10 quality‐filtered reads in at least one of two independent PCR replicates.

In theory, the development of MNP markers requires high‐quality reference genomes—a critical barrier for non‐model organisms, which dominate biodiversity hotspots like the Yangtze River. Nevertheless, our study demonstrates that restriction‐site associated DNA sequencing can bypass the need for complete genomes. By generating sufficient polymorphic loci from RAD‐seq data (Figure 1), RAD‐seq democratises MNP marker development for data‐deficient species—a methodological leap that aligns with global efforts to study undercharacterized taxa. However, RAD‐seq requires bioinformatic expertise for data processing and quality control, and initial sequencing costs may limit adoption in resource‐constrained labs. To enhance accessibility, open‐source pipelines and standardised protocols could mitigate these barriers. Scaling MNP markers to multi‐species panels, as proposed for community‐level biodiversity assessments, is promising but faces challenges. These include potential marker overlap across species, necessitating rigorous specificity testing, and complex primer design to avoid cross‐amplification. Future efforts to develop universal MNP panels could leverage high‐throughput primer optimization and machine learning to streamline marker selection, ensuring scalability while maintaining resolution.

In addition, the dynamic nature of aquatic ecosystems (e.g., rapid DNA degradation, fluctuating environmental conditions) complicates the standardisation of eDNA workflows, risking inconsistent genetic data across studies. To address these challenges, integrating MNP pipelines with optimised eDNA protocols (e.g., rapid on‐site filtration, stabiliser buffers for DNA preservation) might mitigate environmental variability (Lu et al. 2024). For instance, coupling MNP analysis with machine learning‐driven degradation models may allow researchers to statistically correct for DNA fragmentation effects, enhancing cross‐study comparability.

Future research should prioritise two directions: (1) Expanding MNP applications to multi‐species panels, enabling community‐level biodiversity assessments while maintaining single‐species resolution. Such panels could unravel trophic interactions or habitat partitioning patterns in complex ecosystems. (2) Integrating machine learning algorithms with MNP datasets to predict population trajectories under climate change or habitat fragmentation. These efforts would align genetic monitoring with predictive ecology, fostering proactive rather than reactive conservation.

## 5 Conclusion

In conclusion, our study establishes a robust and scalable MNP marker system for *S. prenanti* , achieving high sensitivity, specificity, and resolution in eDNA applications. By addressing limitations of traditional genetic tools, the system provides a powerful framework for biodiversity monitoring and conservation evaluation, including artificial stocking effectiveness in the Yangtze River. These findings underscore the transformative potential of MNP markers for advancing aquatic biodiversity research and guiding evidence‐based conservation strategies globally.

## Author Contributions

Baolong Zhang: validation, formal analysis, investigation, writing – original draft, visualisation, funding acquisition. Wei Jiang: data curation, formal analysis, methodology, software. Zhiwei Fang: formal analysis, methodology, software. Hao Chen, Nan Jiang, and Junfei Zhou: investigation, validation. Renjing Wan: methodology, funding acquisition. Sha Li, Tiantian Li, Lu Cai, Huiyin Song, Lun Li, Lifen Gao, and Lihong Chen: investigation, validation, visualisation. Hai Peng: conceptualization, funding acquisition, project administration, supervision, writing – review and editing.

## Conflicts of Interest

The authors declare no conflicts of interest.

## Supporting information


**Table S1:** Information of DNA spike‐ins.

**Table S2:** Information of MNP markers for *S. prenanti* .

**Table S3:** The reproducibility and accuracy of the MNP marker system.

**Table S4:** Alleles identified in *S. prenanti* and *S. davidi* .

## Data Availability

The datasets supporting this study are available from the corresponding author upon reasonable request. Raw sequencing data from RAD‐seq and MNP‐seq analyses have been deposited in the National Genomics Data Center (NGDC) of the China National Center for Bioinformation (CNCB) under BioProject accession numbers PRJCA037095 and PRJCA037097 (https://ngdc.cncb.ac.cn/).
